# Replication and Analysis of Ebbinghaus’ Forgetting Curve

**DOI:** 10.1371/journal.pone.0120644

**Published:** 2015-07-06

**Authors:** Jaap M. J. Murre, Joeri Dros

**Affiliations:** University of Amsterdam, Amsterdam, The Netherlands; National Scientific and Technical Research Council (CONICET)., ARGENTINA

## Abstract

We present a successful replication of Ebbinghaus’ classic forgetting curve from 1880 based on the method of savings. One subject spent 70 hours learning lists and relearning them after 20 min, 1 hour, 9 hours, 1 day, 2 days, or 31 days. The results are similar to Ebbinghaus' original data. We analyze the effects of serial position on forgetting and investigate what mathematical equations present a good fit to the Ebbinghaus forgetting curve and its replications. We conclude that the Ebbinghaus forgetting curve has indeed been replicated and that it is not completely smooth but most probably shows a jump upwards starting at the 24 hour data point.

## Introduction

This paper describes a replication of one of the most important early experiments in psychology, namely Ebbinghaus' classic experiment on forgetting from 1880 and 1885. We replicated the experiment that yielded the famous forgetting curve describing forgetting over intervals ranging from 20 minutes to 31 days. Ebbinghaus' goal was to find the lawful relation between retention and time-since-learning. This is why he fitted the data to two different functions (a power function, 1880, and a logarithmic function, 1885), as have many theorists since (e.g., [1,2–4]). This papers also includes an analysis—including one with a new model—of the shape of the Ebbinghaus' forgetting curve and its replications. Do the replicated forgetting curves have the same shape, or must we conclude that Ebbinghaus' forgetting curve was idiosyncratic and that quite different shapes may occur?

There is currently an increasing interest in replication studies in psychology, motivated by a growing uneasiness in the community about unreliable findings in psychology. It seems particularly important to try to replicate classic studies that are included in every textbook on cognitive psychology and may also be known by the general public. A good example of this is the classic study by Bartlett [5], which until 1999 had only had unsuccessful replication attempts, until finally Bergman and Roediger [6] succeeded in replicating the basic findings. One of the reasons earlier replications may have failed is because not all details were well-documented in the original study from 1932. The exact instructions, for example, were not included. This may explain why Wynn and Logie [7] had found the forgetting gradient in their experiment to be quite different from the one in Bartlett's experiment. Bergman and Roediger [6] also argue that this may have been caused by certain differences in the study design. Replication of classic experiments, thus, serves the dual purpose of verifying the reliability of the original results and uncovering more precisely how the original experiment was conducted.

It is hard to overestimate the importance of Hermann Ebbinghaus' contribution to experimental psychology. Influenced by the work of the German philosopher Herbart, he was the first to carry out a series of rigorous experiments on the shape of forgetting, which he completed in 1880. The experiment itself was preceded by a period in which he tried out a variety of materials and methods. After having tested himself with tones, numbers, and poem stanzas, he decided that none of these served his purposes. Tones were too cumbersome to handle and too difficult to reproduce for him, he did not find digits zero to nine suitable as basic units for the long-running experiments he envisioned, and the poem fragments he tried to learn (from Byron’s Don Juan) were deemed too variable in the meanings they evoked and therefore likely to cause measurement error [8] (p. 14–17). He, therefore, introduced nonsense syllables, which had more uniform characteristics than existing words or other verbal material. In his later experiments on learning, however, he did verify his results with the Don Juan verses, confirming both his main results on learning and his intuition that the latter stimuli did indeed yield much more variance in the data [9]. Since his introduction of nonsense syllables, a large number of experiments in experimental psychology has been based on highly controlled, artificial stimuli.

In all experiments reported by Ebbinghaus [9], he used only himself as a subject. Single-subject designs are not unusual in memory psychology. Especially in the study of autobiographical memory we find several diary studies based on one person’s personal memories (e.g., [10,11,12]). They have the advantage that there is no inter-subject variability, although they still require hundreds of trials to reduce the variance due to differences in stimuli and other factors. This places a great burden on the subject. Indeed, Ebbinghaus’ forgetting curve is based on seven months of experimenting, often up to three sessions per day. Wagenaar [13] meticulously recorded one daily memory during six years and spent several months recalling these.

A disadvantage of a single-subject design is that it remains unclear what the shape of forgetting would be with other subjects. Are the results universal or did the subject happen to have a memory that was exceptional in some way [14,15]. The generality of the results can be assessed with a faithful replication. There have been a number of—mostly early—replications of Ebbinghaus’s forgetting curve, notably by Radossawljewitsch [16] and Finkenbinder [17], but these authors used a much slower presentation rate of the stimuli of 2 s per stimulus, where Ebbinghaus learned at 0.4 s per stimulus. This was partially the result of the development of devices for mechanical presentation by Müller and colleagues [18,19], who presented materials at a rate one stimulus per second. Slowing down the presentation this much alters the nature of the processing with more time to generate meaningful associations to otherwise meaningless syllables. Though the resulting forgetting curves are clearly of interest to the field, we feel that the slow method of presentation form a large departure from Ebbinghaus’ original study. Also, Finkenbinder’s [17] longest retention interval is 3 days, instead of 31 days and though in the experiment by Radossawljewitsch [16] the retention interval range extends up to 120 days, his design suffers from an uneven distribution of intervals throughout time and time-of-day. Stimuli were learned in order: in the first few days of the study all 5 min intervals were learned, then the 20 min intervals, and so on. Because he did not use a pre-experimental practice phase, the early intervals took longer to learn while the subjects were still getting used to the materials and the procedure; it is likely that this has affected the shape of the forgetting curve reported by him. There are other differences between these two studies and Ebbinghaus’, for example, the degree to which was learned and whether the subjects were allowed to pause between lists.

There are several unanswered questions about Ebbinghaus’ results that formed part of the motivation for us to undertake this replication. His basic stimulus was a ‘row’ of thirteen nonsense syllables, which he studied until he could correctly recall it in the correct order twice in succession. A question that seems pertinent is how stimuli at different serial positions were learned and how these were forgotten over time. Another question is how his measure of choice, namely savings (see below) is related to the nowadays more common measure of percentage correct. Finally, we were interested in the role of interference or fatigue in the course of the experiment.

To help answer these questions, we consulted not only the widely published text of 1885 [9], which was translated into English in 1913 [20], but also an earlier report of 1880 [8]. This is a handwritten manuscript that he submitted for his *Habilitation*, which in Germany is a requirement to be considered for a full professorship. This text (the so called *Urmanuscript* or original manuscript) has been typeset and republished in German in 1983. Even with this additional source, however, we still could not answer the questions above.

For these reasons, we decided to replicate Ebbinghaus’ forgetting experiment. If our replication yielded similar results, this would support the generality of Ebbinghaus’ curve and through a more detailed analysis of our data, we would be able to address the issues above. In the course of preparing for our study, we found that there has been at least one other replication study, namely by Heller, Mack, and Seitz [21]. This study has been published only in German, without an English abstract, and is not easily accessible; at the time of writing, it is not available in electronic format (i.e., it is not available online) and it has never been cited in international journals in English. It is, however, a thorough study and an excellent replication attempt. Where the Ebbinghaus [8,9] texts are unclear about certain details, we have mostly followed Heller et al. [21] as a guideline so that we can also compare our results with theirs. Because we feel this is an important study that has not received the readership it deserves, we will mention more of its details here than we would have had it been more accessible at this point in time.

In 1885, Ebbinghaus introduces the savings measure of learning and memory (it does not appear in this form in his earlier text from 1880). Savings is defined as the relative amount of time saved on the second learning trial as a result of having had the first. Suppose, one has to repeat a list for 25 times in order to reach twice perfect recollection and that after one day, one needs 20 repetitions to relearn it. This is 5 less than the original 25; we can say that on relearning we saved 20% with respect to the original 25 rehearsals (5/25 = 0.2 or 20%). If it takes just as long to relearn the list as it took to learn it originally, then savings is 0. If the list is still completely known at the second trial (i.e., no forgetting at all), then savings is 1 or 100%. Ebbinghaus prefers to express savings in terms of time spent learning and relearning but the principle remains the same. After Ebbinghaus’ publication in 1885, the savings measure remained popular for several decades [16–19,22–24]. Eventually, researchers found the savings method too unreliable compared with other methods of measuring memory [24] and in the following decades it was used much less with some exceptions (e.g., [25]). Later, an important improvement was suggested [26,27], where learning is not to the 100% criterion but to a much lower one, such as 50% correct. These improved versions of the method are used nowadays, for example, when studying forgetting of foreign languages [28–30].

In the following, we will first report our replication experiment. Then, in the Discussion section we will revisit the shape of forgetting, analyze the effects of serial position on forgetting, and investigate what mathematical equations present a good fit to the Ebbinghaus forgetting curve and its replications. Finally, we will study whether there is evidence for a jump at 24 hours in these curves, which some authors have attributed to the effect of sleep.

## The Replication Experiment

The current study was set up to replicate the findings by Ebbinghaus [8,9]. Despite a quite detailed account of his experiment, we found some information to be lacking and we had to estimate or guess these details, as outlined below. Also, we did not have the seven months available that Ebbinghaus invested in the experiment, but we had to accommodate our design to a 75 day period. We nonetheless believe that our experiment is close enough to his to be still called a replication.

There were a few differences between Ebbinghaus’ study, Heller et al.’s [21] replication, and ours. *(i)* Because we were limited in time, like Heller et al. [21], we ran only 10 replications per time interval, instead of the 12 to 45 by Ebbinghaus. This means the variance in our data is larger than in Ebbinghaus’ especially at the longest time intervals; apart from that no systematic differences were introduced. *(ii)* We were not able to experiment at a fixed time of day. Ebbinghaus (1880), who started experimenting in the morning at (A) 10:00, and then sometimes also at (B) 12:00 and usually at (C) 19:00 to 20:00, noticed that there was a difference between these times of the day in his ability to acquire a list. He subtracted 5% for B and 13% for C from the learning times at these hours to normalize the data with respect to time A. Heller et al. [21] were also able to conduct experimental sessions at specific times throughout the day, but they did not find such a time-of-day effect and hence did not implement a correction. *(iii)* Our stimulus material conformed to the phonotactics of the Dutch language and thus differs from both Ebbinghaus and Heller et al. Also, in contrast to both Ebbinghaus and Heller et al. we removed syllables that had too much meaning in order to further balance the level of difficulty of the stimuli. *(iv)* Our subject, J. Dros, was younger than H. Ebbinghaus, who was 29 during his experiments in 1879–1880. The ages of the two subjects in Heller et al. [21] are not given. *(v)* Ebbinghaus [8] gives exact testing dates for each of the short time-intervals but not for the longer ones (24 hours and up). Hence, we do not know exactly when he learned and relearned the lists for the longer intervals. This makes it impossible to calculate the number of interfering lists between learning and relearning. It also makes it nearly certain that our schedule differed from his (and from that of Heller et al. [21] who also do not supply such a schedule).

### Subject

The second author, J. Dros, (22 years, male) was the only subject in the experiment. This experiment was reviewed and approved by the Review Board of the Psychology Department of the University of Amsterdam (see www.lab.uva.nl). The project is filed with case number 2014-BC-3879 (contact is Dr. R.H. Phaf). Consent was implicit as the second author of the paper was also the only subject on which we report. This was also approved by the Review Board. The subject's native language is Dutch, making this the first non-German replication of Ebbinghaus’ forgetting experiment.

### Materials

The learning material consisted of 70 lists. Each list consisted of 104 nonsense syllables, which in turn consisted of 8 ‘rows’ of 13 syllables.

#### Nonsense syllables

Each syllable consisted of 3 or 4 lower-case letters. The structure of a syllable was a lower-case consonant-vowel-consonant (CVC) structure. The consonant of the syllable was always one of b, d, f, g, h, j, k, l, m, n, p, r, s, t, or w. The vowel could be one of e, i, o, u, aa, uu, ee, ei, eu, oe, ie, oo or ui. The double-letters stand for standard Dutch vowels. The last consonant of the syllable was one of f, g, k, l, m, n, p, r, s, or t.

The number of different possible consonant-vowel-consonant combinations on the basis of these letter combinations is 2100 (15 × 14 × 10). Not every possible consonant-vowel-consonant combination was included in the learning material; we removed words that had too much meaning in Dutch, in order to further balance the difficulty of the stimuli. Syllables with meanings in other languages spoken by the subject, such as English and German, were not excluded.

#### Row and list construction

Using the pseudo-random generator of Excel 2010, rows of 13 syllables were constructed. Within a row we did not allow two syllables with the same vowel in direct succession. We also did not allow two identical syllables within one row, but we did allow them in different rows within a single list. When syllables needed to be adjusted we first tried changing only the first or second letter of a syllable until the criteria were met. If this did not suffice, additional letters were changed. The adjustment process was not purely random but was carried out by hand during stimulus preparation by the authors.

### Procedure

The only independent variable in this experiment was the time-interval, which started at the end of learning a list for the first time. The time-interval ended at the beginning of learning a list for the second time. The time-intervals between learning and relearning were the same as Ebbinghaus [8]: 20 minutes, 1 hour, 9 hours, 1 day, 2 days, 6 days and 31 days. For each time interval, 10 lists were learned and relearned (for the 9 hour interval only 9 lists were learned due to unforeseen circumstances).

We need to elaborate on the choice of these time intervals as there is some confusion about the exact length of the shorter retention intervals used by Ebbinghaus. He mentions both 15 min (in 1880 [8]) and 19 min (in 1885 [9]) for the shortest interval, and 63 min and 8.75 hours (525 min) for the longer intervals. He also states that relearning took place “after about one third of an hour, after 1 hour, after 9 hours, one day, two days, six days, or 31 days.” ([20], p. 66). Heller et al. [21] followed the latter intervals, using 20 min, 60 min and 9 hours, etc. We have also used these, given that this seems to have been the intended lengths of Ebbinghaus’ retention intervals. The deviations these values by Ebbinghaus are based on corrections after the experiment.

Ebbinghaus’ shortest interval (‘20 minutes’) is based on almost immediately relearning a list of eight rows and hence the interval depends on how long it took to learn the eight lists. When relearning the lists so soon it takes much less time to relearning them, than the original learning, so that the intervals between learning and relearning of lists is not constant, with List 8 being relearned earliest (e.g., 20 min) and List 1 latest (e.g., 10 min). Ebbinghaus [8] (p. 50) states that the average time is about 15 min and argues that that whereas he does not know exactly how to correct for these variable learning times, the error will be small. We recalculated the average of the times stated on page 51 of the 1880 text and find it to be 1010 s or 16.8 min. Ebbinghaus keeps using the value of 15 min throughout his text from 1880, including for fitting his ‘power function’ equation (see below). The learning and relearning times given in his 1885/1913 volume [20] are the same as in 1880 [8], but to each interval he has now added 88 s for reasons that are not made clear. The average of these learning intervals then becomes 18.3 min. Given that he also remarks that for the shortest interval “relearning of the first series of a test followed almost immediately or after an interval of one or two minutes upon the learning of the last series of the same test” ([20], p. 66) may explain rounding 18.3 min to 19 min, the value used throughout the text from 1885. In general, it seems he made more or less intuitive corrections for the variable learning times and changed his mind from 1880 to 1885 about the most appropriate method to approach this. We have used 19 min, 63 min, and 8.75 hours in the graphs and tables (and fits), for Ebbinghaus’ data. For the other data sets, we use 20 min, 1 hour, and 9 hours.

#### Measurement of repetitions and time

The main measurement was the number of repetitions needed to correctly reproduce the syllables in a row in the correct order.

For the forgetting curve experiment, Ebbinghaus [8] learned until twice correct, but in later experiments switched to once correct [9], because he found there to be no essential difference in the outcomes. We chose to also learn to once correct. Heller et al. [21] (p. 8) seem to be using the once-correct criterion as well but this is not made entirely clear.

Ebbinghaus [8] uses elapsed time to calculate the number of repetitions, because he finds keeping count too distracting. Heller et al. [21] use a chain with wooden, colored beads, much like a rosary to keep count. We found that word processing software (Microsoft Word) was handy to keep track of the number of repetitions. During learning, each single repetition of a row was counted by pressing the button ‘1’ on the keyboard at the beginning of every single repetition of a row. At the end of learning a list, the total number of 1’s for each row was counted and entered into the database.

We also measured the time in seconds needed to learn a list. A clock was shown on the computer screen during the task and we recorded the begin times and end times of learning a list. Subtracted from the time were the pauses of 15 s between two rows (cf. [8], p. 19). When relearning a list, the extra time (15 s) introduced by the voice recording of the first retrieval attempt was subtracted from the total relearning time.

#### The practice phase and experimental phase

Following Ebbinghaus, we preceded the experimental phase of the experiment with a practice phase to prevent as much as possible general learning effects due to growing experience with the task and materials. The practice phase took place between 08-11-2011 and 29-11-2011. A total of 14 lists was learned and relearned after 20 minutes (Heller et al. [21] relearned lists after one hour). After these, a further 19 lists were learned only (i.e., not relearned later) for additional familiarization with the task.

In the experimental phase, which took place between 01-12-2011 and 13-02-2012, a total of 69 lists was learned and relearned (9 for the 9 hour interval). The total time spent on data collection in the experimental phase amounted to about 70 hours. We distributed the ten lists for each time interval as much as possible over the whole experimental period. Due to the limited time available to run the whole experiment, we were not able to achieve this for the 31 days time interval condition, so that we decided to learn these lists near the beginning of this experimental period.

#### List learning phase

All lists were printed on paper (black ink, font ‘Calibri’, 11 points) (eight rows per page; a row was actually printed in a column format for easier studying). The non-studied rows were covered by sheets of paper. The subject was seated behind a desk in a quiet room. The main goal was to learn a list as quickly as possible, to learn each row until it could be reproduced correctly once.

Following Ebbinghaus [8] (p.18), the syllables were softly spoken from the first syllable to the 13^th^ syllable at a constant speed of 150 beats per minute. The repetition of a row took 5.2 s on average. Our subject preferred to speak the syllables in a jambus-like manner, where syllables were paired so that the emphasis always was on the second syllable (i.e. wes-hóm, niem-hág, etc…). The last syllable (13^th^ syllable) was not paired to another syllable and was not spoken with an emphasis. Here, we use an approach similar to Heller et al. [21] and not to Ebbinghaus, who prefers a ¾ rhythm, stressing the first syllable in each group of three (this is in fact the reason he gives for his preference for rows of length 10, 13, or 16 syllables, see Ebbinghaus [8], p. 19).

During the learning phase, the subject had a continuous choice to either read or reproduce the syllables. Towards the end of the learning process, occasional attempts were made to produce an entire row by heart. When there was a moment of hesitation during such ‘blind’ reproduction, the rest of the list was read (i.e., not blind) to the end. Blind reproduction always started with the first syllable of a row. Rows were not learned in parts. Each time the 13^th^ syllable had been reached and the row still contained errors, it was read again from the beginning. After having learned one row and before starting the next one, there was an interval of 15 sec. The interval served as a moment of rest and pause. All of this was aimed at following Ebbinghaus as closely as possible. Each row was thus learned to a 100% correct criterion before moving to learning the next row.

During the pre-pretraining phase, a metronome was used at first to achieve a recitation rate of 150 beats, but this was found to be too intrusive and distracting. Eventually, the rhythm was internalized and the metronome was only used for occasional rate checks. During the experimental phase, it was not used. After each rehearsal of a row, there was a little transition-pause of about 3 beats to take a breath before the next repetition of the row.

**Fig 1:**
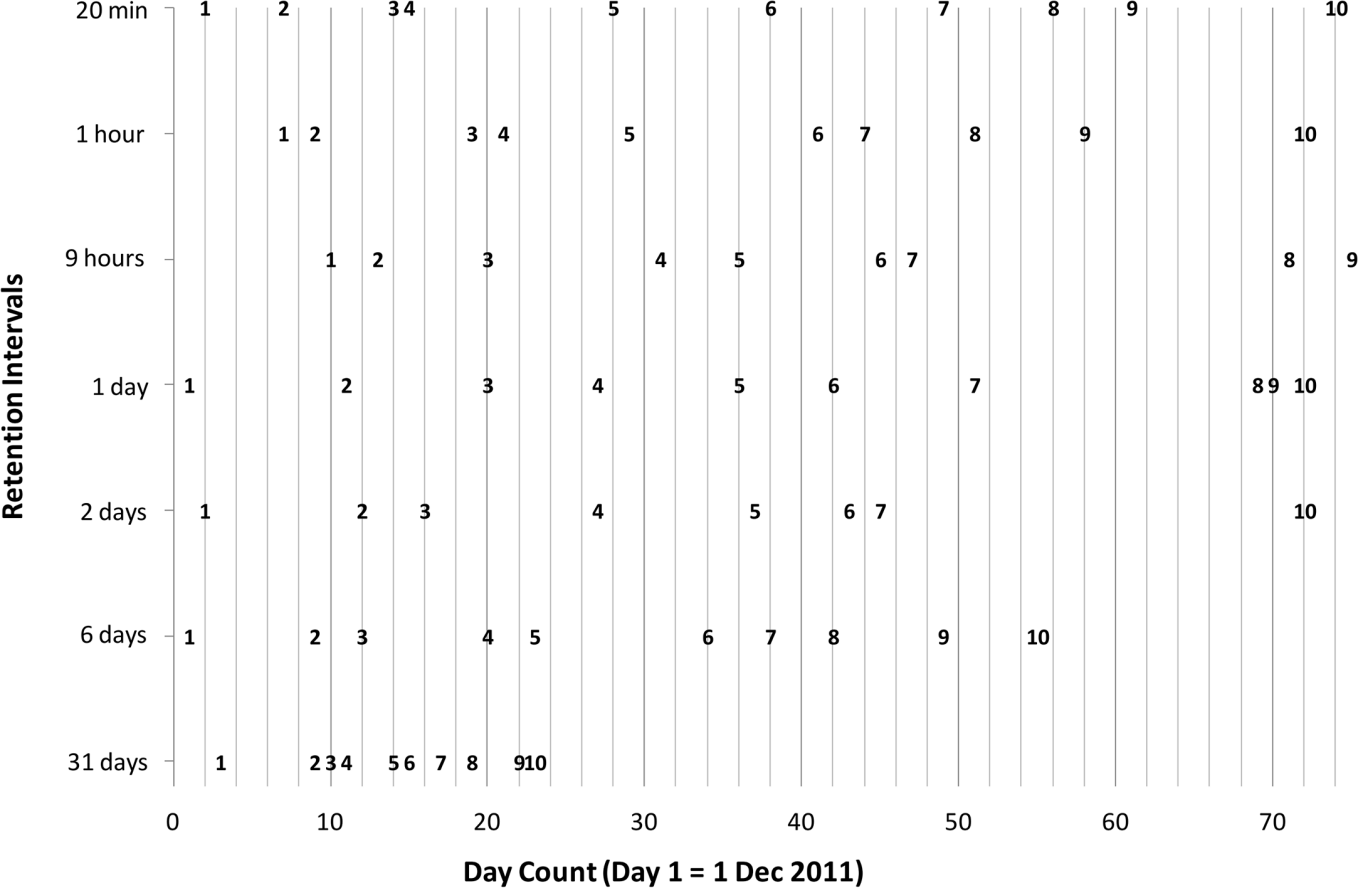
Learning schedule during 2011–2012 for all lists, where labels in bold indicate when each of the lists 1 to 10 was first learned for each retention interval. Relearning times are not shown but can be derived by adding the retention interval (e.g., 6 days).

Learning of a list was considered complete, if all rows had thus been learned in order. The retention interval was started at the time a list had been learned. On most days two or three lists were learned or relearned with a maximum of four. The full learning schedule is given in Fig 1.

#### Relearning phase

We added one additional measurement to Ebbinghaus’ procedure: the number of correct syllables at first reproduction of a row at relearning, not necessarily at the right location. We recorded the recall of the first time a row was relearned with an Olympus WS-450s voice-recorder. After the last relearning session of the experimental phase, the sound files were transcribed and scored.

Rows were relearned in the same order as during original learning. During relearning, the subject was seated at a desk with a computer. A word processing program was opened on the computer and a clock was visible on the screen. A sheet of paper with a list printed on it was laid in front of the subject with only the syllables from the row to be learned visible. The other rows were covered by a piece of paper. During learning, the subject used the ‘1’ button on the keyboard to count the number of repetitions. Following Ebbinghaus [8] (p. 19), after successful relearning there was a 15 second pause. Then, the row learned last was covered, a next row uncovered and the procedure was repeated.

Relearning of a row started with turning on the voice recorder. Then the row was read once as described above. Twenty seconds after turning the voice recorder on, the subject stopped recall attempts and turned the voice recorder off. After that, relearning continued in the usual fashion. This procedure was repeated for every row in the list. After a list had been relearned, the audio file of the recording was saved on a computer.

## Results

### The forgetting curve

The main objective was to replicate Ebbinghaus’ famous forgetting curve. The average number of repetitions is given in Table 1 and the number of seconds spent on learning and relearning each list, with the calculated savings scores, is given in Table 2. The raw data of this experiment are freely available online at the website of the Open Science Foundation (URL: https://osf.io/6kfrp/). To see whether there were differences between the time-intervals in the average number of repetitions at first learning, a one-way independent ANOVA with the average number of repetitions per list as the dependent variable and the time-interval as the independent variable. There was no significant effect for the time-interval, F(6, 69) = 0.691, p = 0.658. This means that the average number of repetitions per list did not differ significantly per time interval, indicating there were no randomization confounds.

**Table 1:** Average number of repetitions until once correct.

	*n*	*Learning*	*SD*	*n*	*Relearning*	*SD*
***20 min***	10	30.77	2.90	10	16.26	2.27
***1 hour***	10	30.64	2.28	10	19.22	1.56
***9 hours***	9	31.07	2.08	9	22.48	2.67
***1 day***	10	31.22	2.34	10	21.33	2.11
***2 days***	10	31.42	2.49	10	24.19	2.30
***6 days***	10	31.21	3.19	10	25.97	3.37
***31 days***	10	29.44	2.26	10	28.23	3.48

**Table 2:** Time spent learning (session S1) and relearning (session S2) for each list with savings (Q) by Dros.

	*20 min*			*1 hour*			*9 hours*			*1 day*			*2 days*			*6 days*			*31 days*		
*List*	*S1*	*S2*	*Q*	*S1*	*S2*	*Q*	*S1*	*S2*	*Q*	*S1*	*S2*	*Q*	*S1*	*S2*	*Q*	*S1*	*S2*	*Q*	*S1*	*S2*	*Q*
*1*	1405	670	0.523	1690	1280	0.243	1815	1240	0.317	1670	1105	0.338	1710	1195	0.301	1780	1370	0.230	1480	1380	0.068
*2*	1840	1210	0.342	1790	1330	0.257	1780	1350	0.242	1840	1325	0.280	1635	1340	0.180	1605	1560	0.028	1680	1450	0.137
*3*	1830	1100	0.399	2070	1235	0.403	1935	1350	0.302	1930	1205	0.376	1950	1580	0.190	1870	1545	0.174	1770	1530	0.136
*4*	2180	960	0.560	1875	1130	0.397	1525	975	0.361	1740	1365	0.216	1935	1440	0.256	2020	1805	0.106	1440	1510	-0.049
*5*	1800	840	0.533	1775	1245	0.299	1770	1275	0.280	1875	1410	0.248	1830	1500	0.180	2090	1785	0.146	1650	1760	-0.067
*6*	1815	1345	0.259	1765	1170	0.337	1815	1335	0.264	1710	1215	0.289	2130	1485	0.303	1740	1585	0.089	1890	1785	0.056
*7*	2040	1110	0.456	1680	1125	0.330	1635	1220	0.254	1905	1325	0.304	1890	1440	0.238	1710	1350	0.211	1815	1745	0.039
*8*	1725	865	0.499	1905	1250	0.344	1845	1380	0.252	2095	1235	0.411	2085	1460	0.300	2025	1665	0.178	1910	1505	0.212
*9*	1935	1320	0.318	1805	1155	0.360	1950	1585	0.187	1860	1290	0.306	1740	1335	0.233	2100	1415	0.326	1490	1260	0.154
*10*	1830	1235	0.325	2065	1325	0.358				1980	1275	0.356	1695	1375	0.189	1890	1275	0.325	1710	1395	0.184
*Average*	1840	1066	0.421	1842	1225	0.335	1786	1301	0.271	1861	1275	0.315	1860	1415	0.239	1883	1536	0	1684	1532	0.090

**Fig 2:**
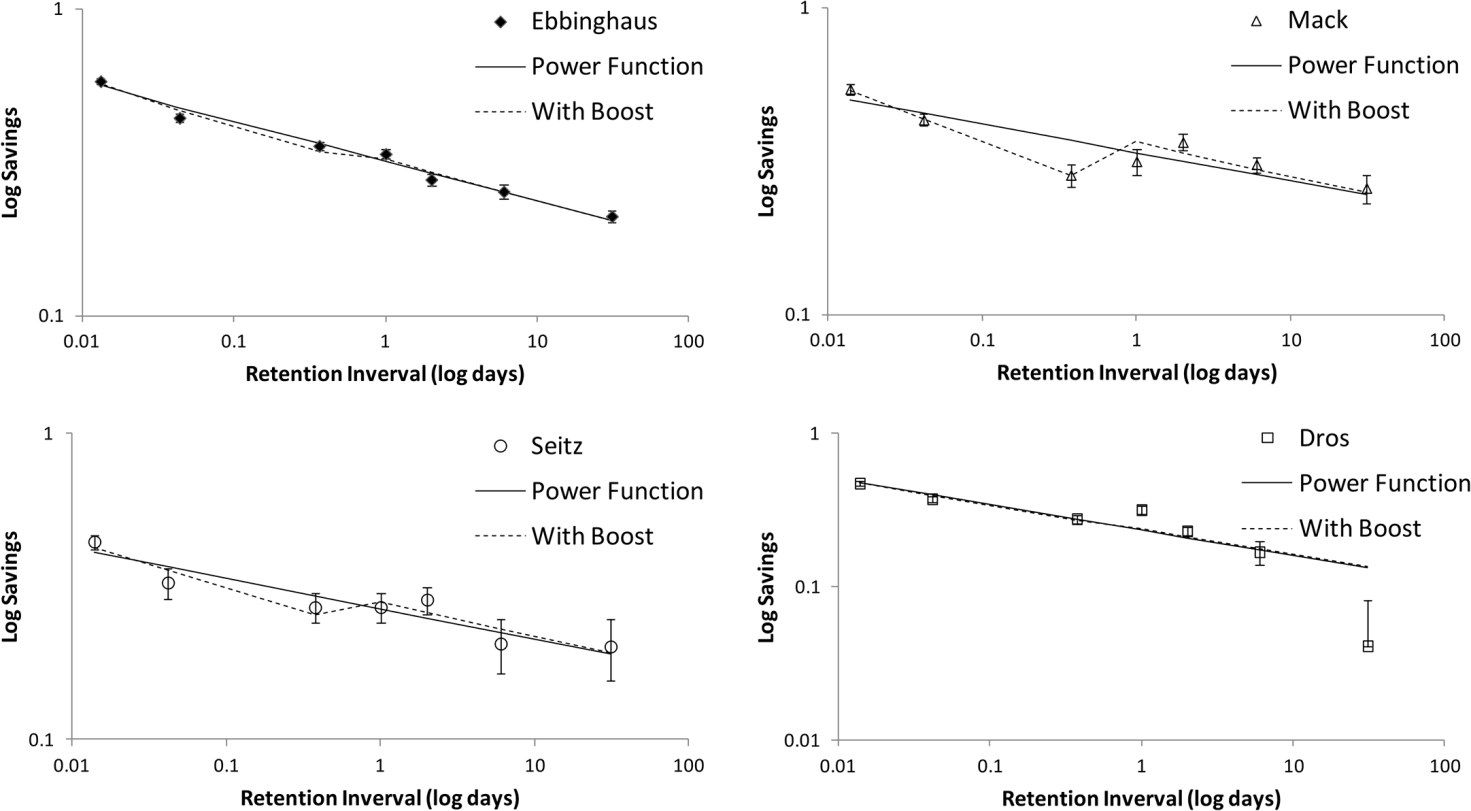
Four loglog graphs with savings as a function of retention interval with fitted power function curves and curves with best fitting power functions with boost at 1 day (see text for an explanation).

**Fig 3:**
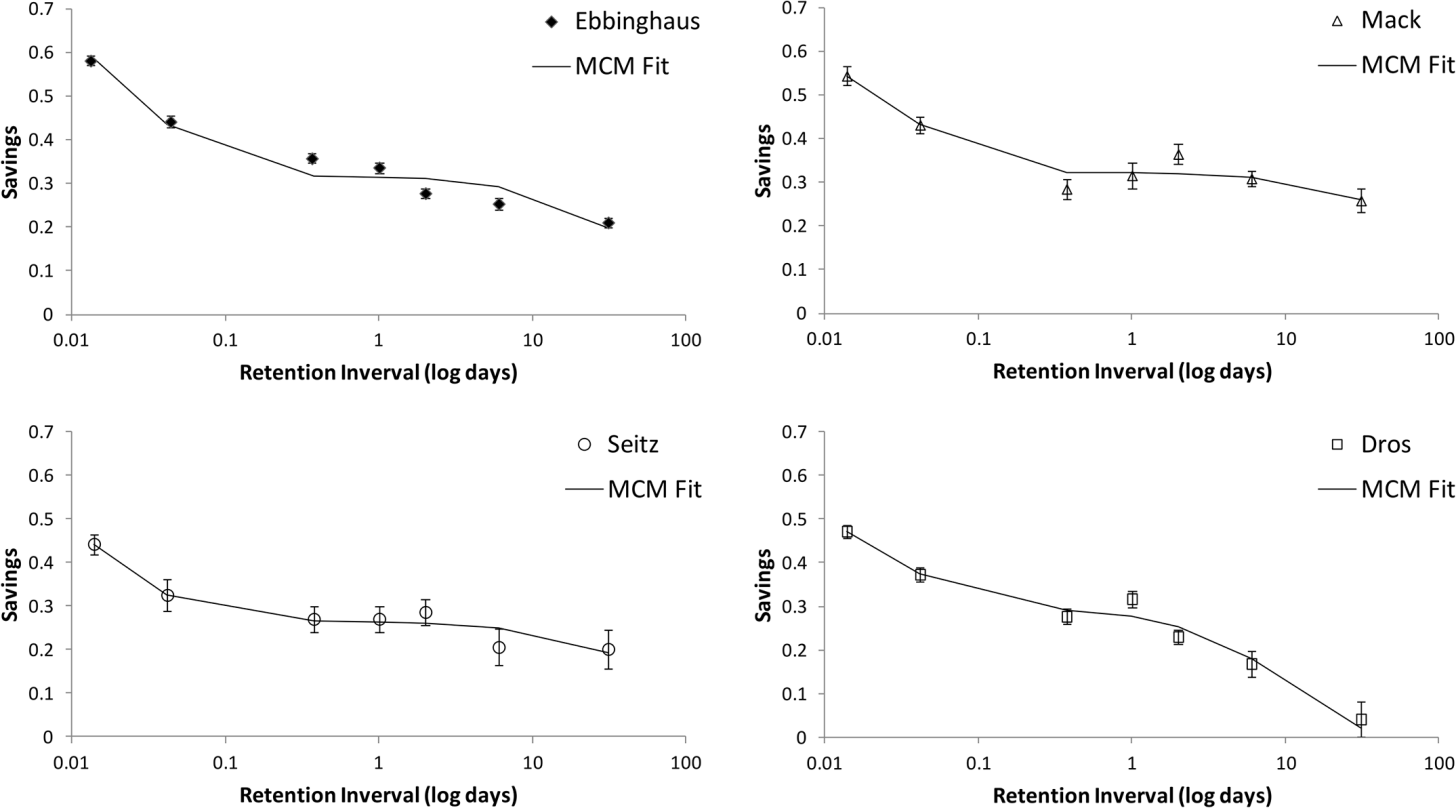
Four log graphs with savings as a function of retention interval with best-fitting Memory Chain Model retention functions (see text for an explanation).

**Fig 4:**
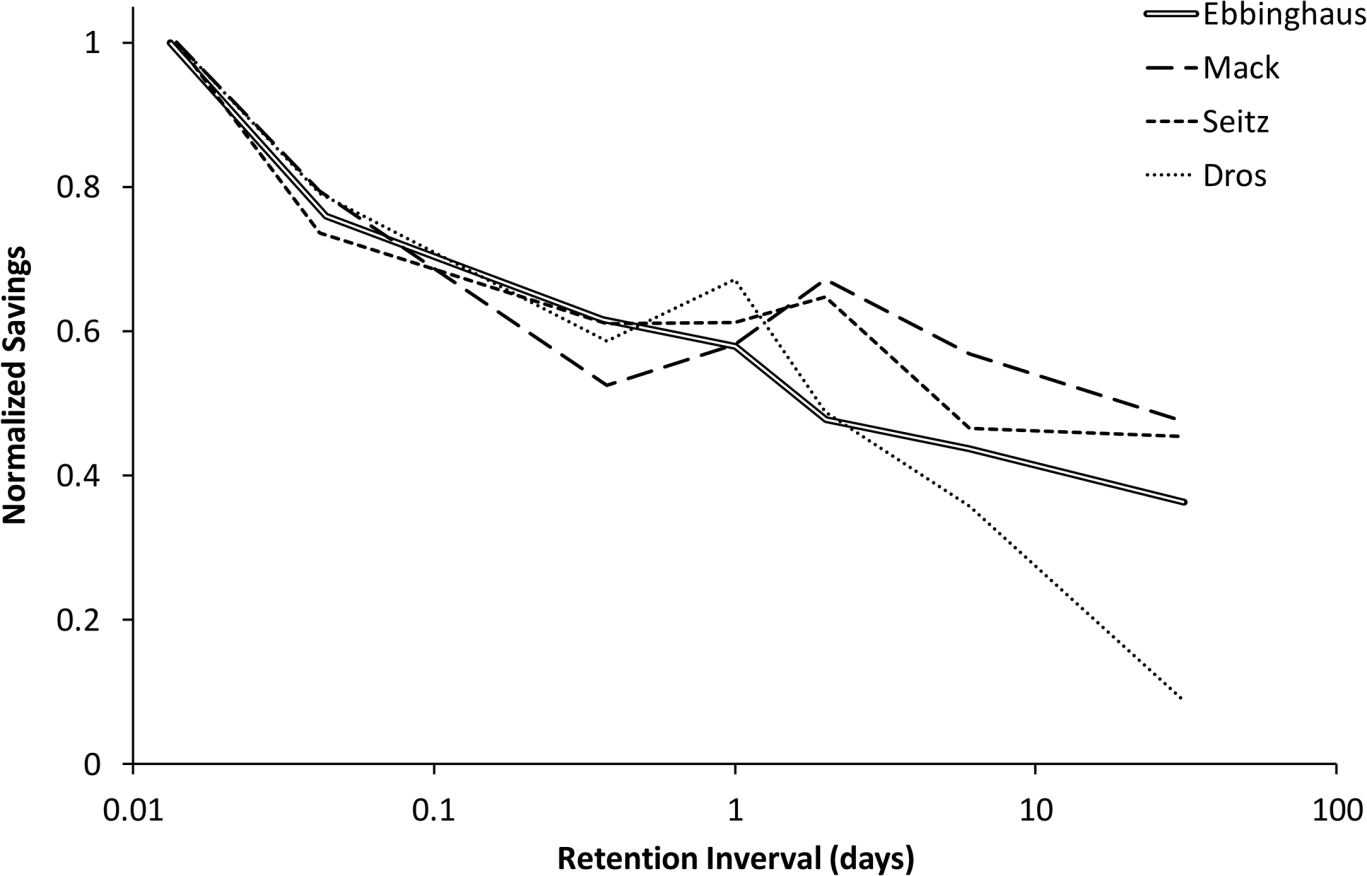
Normalized savings scores as a function of retention interval on a logarithmic scale, rescaled so the first data point is 1.0 for all curves.

Savings scores (based on time in s) are compared with those of Ebbinghaus [8], and Mack and Seitz [21] in Table 3 and plotted with error bars in Fig 2 using loglog coordinates and in Fig 3 using log coordinates only for the time axis. Despite the fact that the original experiment dates from 1880 and replications were done over a century later, and despite the fact that our replication was carried out in a Dutch language context, the four forgetting curves share many characteristics. To facilitate a direct comparison we have overlaid the four curves in Fig 4 where we have normalized the savings scores such that the first data point (at 20 min) was always equal to 1.0. Given expected individual differences, we find the resemblance of the four graphs remarkable. The greatest deviation is by Dros at 31 days; his savings score is much lower that any of the other three. We can only speculate at the reason for this. It may be that this subject simply has more long-term forgetting. Another explanation bears on the fact that the lists for the 31 day interval were all learned early in the experiment (see Fig 1) and learning times were shorter at the beginning, due to greater initial enthusiasm, less pro-active interference, or yet another reason. It is also possible that the low savings score on the 31 day point is an effect of the relatively short time period in which initial learning took place for the 31-day data point (i.e., massed learning); learning for other intervals was more widely spaced. We were forced to place the learning sessions at the beginning because of the limited time available by the subject for data collection. Ebbinghaus could spread his sessions of a seven-month interval, though we do not know the exact schedule for the intervals past 9 hours, nor do we know anything about the schedule followed by Mack and Seitz. Finally, we considered where the number of intervening lists had influenced retention at the 31-day-point. For this time point, the number of intervening lists varied from 23 to 33. Our analysis, however, revealed virtually no relationship with savings score as a function of intervening lists (*R* ^2^ was about 8.4%). Our data did not allow us to further disentangle the effect of number of intervening lists versus time because list number and time were too strongly confounded in our design.

**Table 3:** Savings for Ebbinghaus ([8], p. 56, see Note 1 for the comments on the intervals 20 min, 1 hour and 9 hours), Mack and Seitz [21], and Dros (this paper).

	Ebbinghaus	Mack	Seitz	Dros
**20 min**	0.582	0.544	0.442	0.472
**1 hour**	0.442	0.432	0.325	0.373
**9 hours**	0.358	0.285	0.270	0.276
**1 day**	0.337	0.316	0.270	0.317
**2 days**	0.278	0.365	0.286	0.230
**6 days**	0.254	0.309	0.205	0.168
**31 days**	0.211	0.258	0.201	0.041

**Fig 5:**
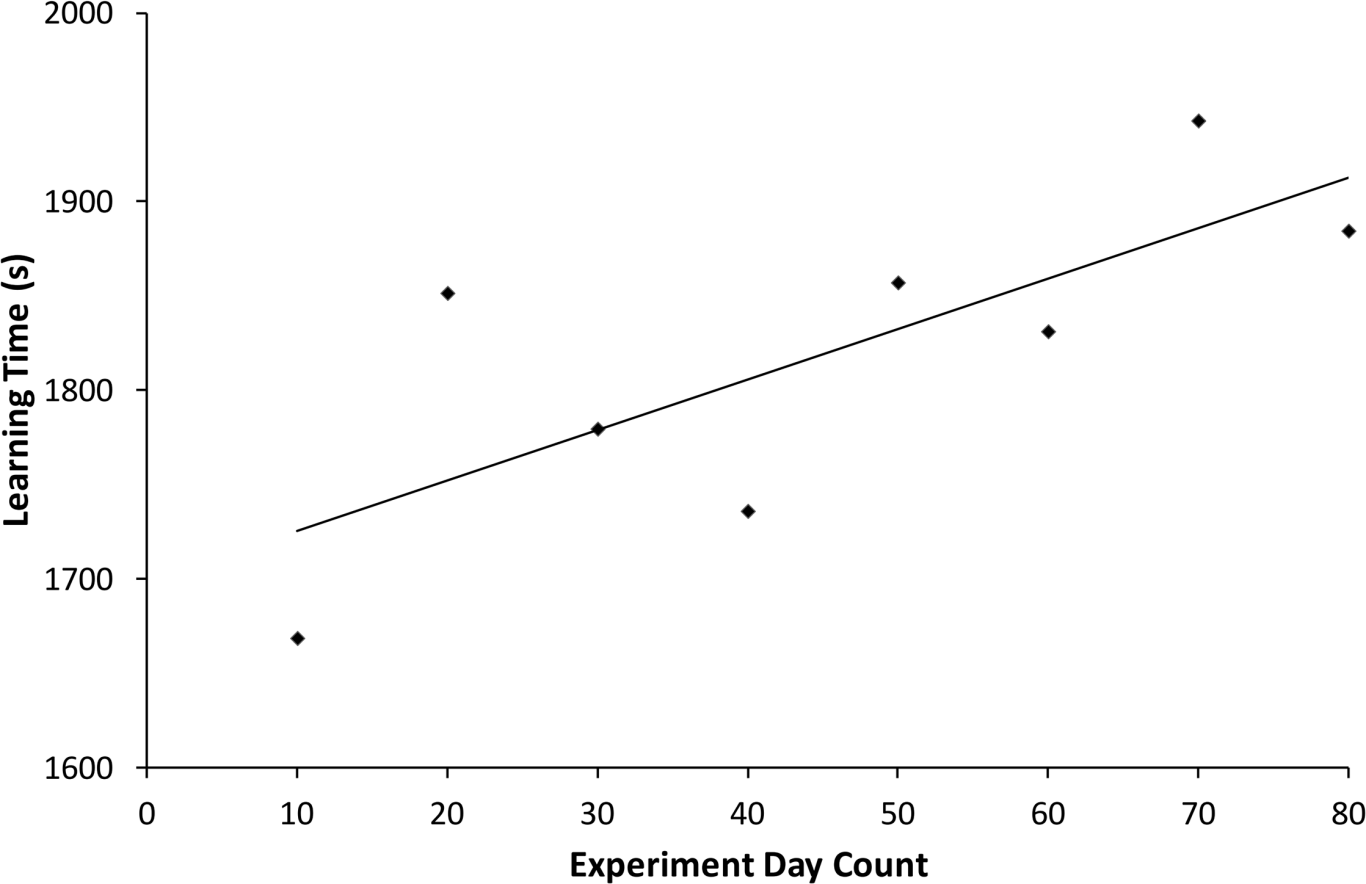
Learning time per list as a function of day of experiment with a fitted straight line.

We found a gradual increase in learning time throughout the course of the experiment as can be seen in Fig 5, where averaged learning time in s has been plotted for consecutive ten-day periods (‘bins’). In the course of the 75 days of the experimental phase there was an average increase in learning time of 2.67 s per day for a list (this linear regression explained 56.18% of the variance). If we correct for this steady increase, which mostly affects the 31 day interval, the corrected savings measure would be 0.137 for the 31 day interval instead of 0.0410. This, however, is still well below the values for the three others, which are in the 0.20 range. This steady increase in learning time may be due to pro-active interference or fatigue. Ebbinghaus [8] and Heller et al. [21] do not report or analyze this.

We also analyzed the false alarms and correct answers measured on first relearning. We did a one-way independent ANOVA with the number of false alarms per row as the dependent variable and the retention interval as the independent variable. There was no significant effect for the time interval, *F(6*, *512) = 0*.*753*, *p > 0*.*608*, indicating that the number of false alarms was not significantly different for the time-intervals. A one-way independent ANOVA with the number of hits per row as the dependent variable and retention interval as the independent variable, however, yielded a significant effect for the time-interval, *F(6*, *512) = 3*.*85*, *p < 0*.*01*, which we will analyze further in the next section.

### Serial position effects

**Fig 6:**
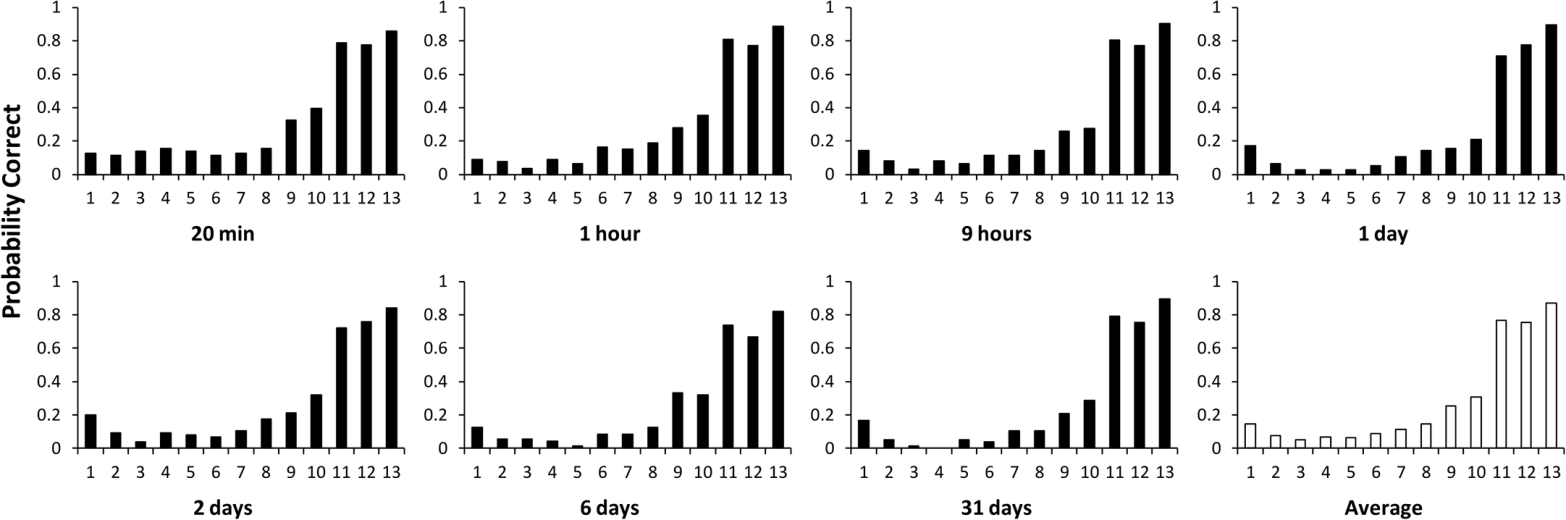
Serial position for correct relearning scores for each retention interval and for the average of all retention intervals (see text for an explanation).

In Fig 6, we have plotted the average serial position curves for each retention interval and for the grand average. Even if a correct syllable was not mentioned at the correct position, it was still scored as correct for its intended position (this was rare and had only a small effect on the data). Fig 6 shows clear serial position curves for all retention intervals.

**Fig 7:**
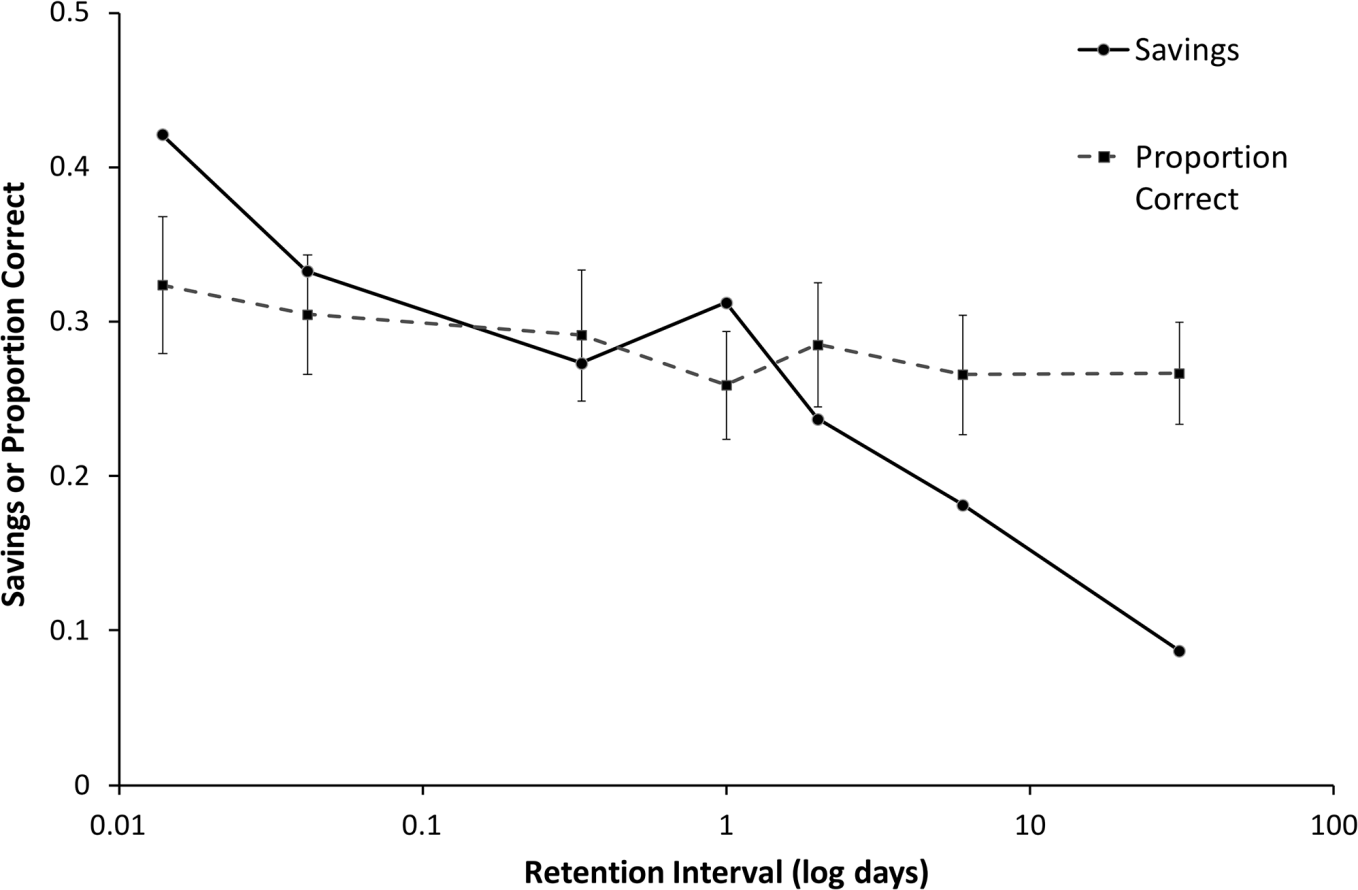
Proportion correct as a function of retention interval on a logarithmic scale. (a) Proportion correct, averaged over all serial positions, shown with Dros’ savings scores for comparison. (b) Proportion correct curves for different groups of serial positions and for the average over all 13 positions.

In Fig 6, it also seems that there is more forgetting with time in the middle positions. In Fig 7A, overall forgetting is shown, where the average of all positions is shown for each retention interval. Though there is forgetting, these curves are much shallower than the savings curves, which are shown as well for comparison. In Fig 7B, forgetting is shown for four groups of serial positions, indicating that indeed there is virtually no forgetting in the final positions 11 to 13. A regression line is nearly horizontal for positions 11–13 (slope -0.000183) and 1–2 (slope is -0.000112) with the largest decrease over time found in positions 3–8 (slope is -0.0114) and 9–10 (slope is -0.0119). The averaged curve has a slope of -0.00930, with all slopes calculated over the untransformed scores (i.e., not on a logarithmic scale).

## Discussion

We believe that we may conclude that our attempt to replicate Ebbinghaus’ classic forgetting was successful. We were able to follow his method quite closely and the resulting curve is very similar to both that of Ebbinghaus and that of the two subjects in an earlier German replication [21], with the exception of the savings value at 31 days, which in our case is much lower than the others. The latter difference remains even if we correct for increased learning time over the course of the experiment. It is possible that with Ebbinghaus and Heller et al. there were far fewer intermediary lists learned between learning and relearning and hence much less interference. Unfortunately, this information is not available, so this must remain speculation.

### Effects of serial position on forgetting

Ebbinghaus does not say anything about serial position curves or indeed about the order in which he acquired the syllables. Our data allow us to say a little more about this. When interpreting the serial position scores in Fig 6, one has to bear in mind the nature of the savings methods with lists of nonsense syllables. With Ebbinghaus-type relearning, a row is always first studied before it is relearned. Savings experiments are very different from normal memory retention experiments where the subject learns something at after some time interval is tested for retention. With savings, the retention measurement itself consists of relearning the original material in repeated recall trials each of which is preceded by prior exposure to the stimulus materials. In theory it is, therefore, possible in extreme cases that there are subjects who can learn a row of 13 syllables in a single learn-recall trial but who subsequently always forget the learned materials in say 10 min. By conventional (non-savings) retention tests, these subjects would be reported as have 0% retention past 10 min, but these same subjects would show 100% savings for all retention intervals past 10 min because when testing their savings performance they would go through the exposure-learn sequence, always (re)learning the materials immediately: at t = 0, they take 1 trial to learn the material and at t = 1 day they also take 1 trial, showing perfect savings (i.e., seemingly no forgetting). Thus, when stimuli are very easy to learn true forgetting becomes impossible to measure with the savings method, because original learning will be at ceiling (one-trial learning) as will be relearning after a time-interval (again: one-trial learning), no matter how long the interval.

Here, it seems that the first two and the last three syllables were very easily learned (and relearned), probably because of primacy and recency effects. This effect was also noticed by other early researchers who adapted Ebbinghaus’ method (e.g., [17]). Relearning the harder parts of a list, in particular the middle syllables, benefits most from recently having learned these sometime before (i.e., before the relearning phase of the savings experiment), as is evident from the savings scores. The discrepancy between savings and recall or recognition has also been found by other authors (e.g.,[24,31,32,33]) and appears here as a function of serial position.

The fairly evident primacy and recency effects also suggest that the despite Ebbinghaus’ efforts to construct equivalent stimuli, there is a great variation in how well they eventually were learned: During the first phase (t = 0), stimuli in the first and last positions were very easily and hence very well learned, while those in the middle were learned much less. The effect seems quite constant, however, so that it need not affect the validity of the shape of the forgetting curve, but we should be aware that its shape is based on a combination of very well learned items and just barely learned items. In that sense, the forgetting curve by Ebbinghaus is an average over different forgetting curves of items in various serial positions, which have been learned to varying degrees. This in itself may explain part of the characteristic shape of the curve, which we will explore in the next section.

### Curve fitting

Hermann Ebbinghaus [8] was the first to try to find a mathematical equation that describes the shape of forgetting. Many researchers who used his method have followed suit, also trying to summarize the forgetting curve in a concise equation. In his first manuscript, from 1880, Ebbinghaus proposes the equation *x* = [1 − (2/*t*)^0.099^]^0.51^, where *x* equals 1 minus savings at time *t* (in min). It is of some interest that Ebbinghaus [8] (p. 57–63) puts the entire section in which he fits this equation to the data between square brackets, making it an aside: something that is also interesting but not belonging to the main text. Nonetheless, he gives a well-motivated derivation based on a differential equation of a gradually slowing forgetting process. Interesting is that in the write-up of the experiment in 1885 [9], the equation has been changed to the very different one, which has been become generally known as the ‘Ebbinghaus Forgetting Equation’, rather than the first one, namely *Q*(*t*) = 1.84 / ((log *t*)^1.25^ + 1.84), where the log is taken with base 10. This equation is lacking a derivation and Ebbinghaus remarks on it: “Of course this statement and the formula upon which it rests have here no other value than that of a shorthand statement of the above results which have been found but once and under the circumstances described. Whether they possess a more general significance so that, under other circumstances or with other individuals, they might find expression in other constants I cannot at the present time say.” [20]

We are now in a better position to verify Ebbinghaus’ question about the general significance of his equation by fitting his equations to the other data. The results are given in Table 4 and were obtained with a nonlinear fitting procedure in *Mathematica* 9 (the Mathematica code is available from the author). We calculated several goodness-of-fit measures including the Akaike Information Criterion or *AIC* [49,58], which contrary to, for example, variance explained (*R* ^2^) or sum of squared differences (*SSD*), takes into account (and penalizes for) the number of free parameters. It also allows a comparison of the goodness-of-fit of different models, even if they have different numbers of parameters. Lower values indicate better fit, where a difference of more than 2 is seen as a meaningful difference in goodness-of-fit [49].

**Table 4:** Fits of two equations proposed by Ebbinghaus in 1880 and 1885 to data from his own study and from three replication studies. See text for the meaning of the parameters. SSD is the sum of squared differences between data and fitted curve, R^2^ is proportion variance explained, and AIC is the Akaike Information Criterion. To stay close to Ebbinghaus’ own estimates, the parameters are fitted for time expressed in minutes.

	*Ebbinghaus*	*Mack*	*Seitz*	*Dros*	*Average*
*Ebbinghaus 1880 ‘Power’ Function*					
*μ*_1_	0.523	0.325	0.248	0.516	
*a*_1_	0.101	0.0518	0.0525	0.14	
*SSD*	0.00224	0.0107	0.0043	0.0177	0.00871
*R* ^2^	0.998	0.989	0.993	0.972	0.988
*AIC*	-30.5	-19.5	-26	-16	-23
*Ebbinghaus 1885 ‘Logarithmic’ Function*					
*μ*_1_	1.8	1.34	0.9	1.36	
*a*_1_	1.21	0.873	0.82	1.34	
*SSD*	0.00218	0.00976	0.00403	0.0212	0.00928
*R* ^2^	0.998	0.99	0.993	0.966	0.987
*AIC*	-30.6	-20.2	-26.4	-14.7	-23.

In the table, we see that in the case of his power function from 1880 [8], Ebbinghaus' calculations, carried out by hand, were quite close the computer-optimized parameter values: he found values 0.51 and 0.099 for the parameters, whereas we found 0.523 and 0.101, respectively. For the logarithmic function from 1885 [9], we also found similar parameters to those parameters Ebbinghaus reported: 1.8 and 1.21 for his values of 1.85 and 1.25 respectively.

The goodness-of-fit of his functions is quite good, in both cases explaining 98.8% of the variance (*R* ^2^) for his own data. The equation from 1885 has a slightly smaller *SSD* value (i.e., fits better), which in fact is the lowest value for an individually fitted curve we obtained (also see below and Table 5). Though the equations found by Ebbinghaus fit his own data very well, they do not always fit the other curves well, with especially Mack and Dros showing a relatively bad fit on these ‘classic’ equations. This is perhaps not concluded from the variance explained (*R* ^2^), which is very high for all studies, but if we base our judgment on the *AIC* we observe large differences where the *AIC* for the Ebbinghaus data is almost twice as low as on the Dros data. This suggests that the general applicability of Ebbinghaus equations may be lacking. We further investigate this by comparing Ebbinghaus’ functions with some other functions that have been proposed in the literature.

**Table 5:** Fits of a number of equations to data from Ebbinghaus and replication studies. See text for the meaning of the parameters. SSD is the sum of squared differences between data and fitted curve, R^2^ is proportion variance explained, and AIC is the Akaike Information Criterion. The parameters are fitted for time expressed in seconds.

	*Ebbinghaus*	*Mack*	*Seitz*	*Dros*	*Average*
*Power Function*					
*μ*_1_	1.4	0.965	0.822	1.56	
*a*_1_	0.13	0.0926	0.099	0.167	
*SSD*	0.00285	0.0129	0.00523	0.0163	0.00932
*R* ^2^	0.997	0.987	0.991	0.974	0.987
*AIC*	-28.8	-18.2	-24.5	-16.6	-22
*Power Function with Boost*					
*μ*_1_	1.65	2.13	1.27	1.67	
*a*_1_	0.152	0.194	0.155	0.176	
*Boost*	0.0303	0.131	0.0631	0.011	
*SSD*	0.00232	0.00355	0.0031	0.0162	0.00628
*R* ^2^	0.998	0.996	0.995	0.974	0.991
*AIC*	-28.2	-25.2	-26.2	-14.6	-23.6
*Summed Exponential Function*					
*μ*_1_	0.383	0.315	0.304	0.262	
*a*_1_	0.000319	0.000296	0.000457	0.000353	
*μ*_2_	0.321	0.323	0.266	0.3	
*a*_2_	1.79E-07	7.99E-08	1.22E-07	1.00E-06	
*SSD*	0.00469	0.00356	0.00276	0.00295	0.00349
*R* ^2^	0.995	0.996	0.995	0.995	0.996
*AIC*	-21.3	-23.2	-25	-24.5	-23.5
*MCM Exponential Function*					
*μ*_1_	0.704	0.639	0.57	0.563	
*a*_1_	0.000319	0.000296	0.000457	0.000353	
*μ*_2_	0.000145	0.00015	0.000213	0.000188	
*a*_2_	1.79E-07	7.99E-08	1.22E-07	1.00E-06	
*SSD*	0.00469	0.00356	0.00276	0.00295	0.00349
*R* ^2^	0.995	0.996	0.995	0.995	0.996
*AIC*	-21.3	-23.2	-25	-24.5	-23.5

Ebbinghaus function from 1880 is a type of double power function. A normal power function is described by the equation $Q(t)={\left(1+\mu_{1}\;\text{t}\right)}^{-a_{1}}$, where *Q*(*t*) is savings at time *t* and *μ*_1_ and *a*_1_ are parameters. The latter equation has been proposed by several authors to describe the time-course of forgetting (e.g., [1,3,4,34]). The forgetting mechanism typically associated with a power function is a constant slowing down of the forgetting rate with time (cf. Ebbinghaus' account from 1880 mentioned above). Whereas this is certainly a viable mechanism of forgetting, it can be proven mathematically that (spurious) power functions may emerge from averaging over different subjects or items [35,36]. This has also been shown in simulations considering a wide range of circumstances [37,38]. As argued in the previous section, the forgetting curve (also) averages over items that have been learned to various degrees, due to their serial position. There are, therefore, several reasons to expect and consider the power function.

The goodness-of-fit of the simple power function to our data is given in Table 5. As can be seen, the fit to Ebbinghaus’ data is still impressive, though somewhat less good than either of his own equations. The goodness-of-fit of the power function, as expressed by the *SSD*, averaged over all four subjects’ is comparable to the Ebbinghaus 1885 ‘logarithmic’ function and it is somewhat worse than his 1880 ‘power’ function. The *AIC* is slightly worse, but probably not meaningfully so as the difference in *AIC* measures is only 1.

Heller et al. [21] also fitted the Ebbinghaus 1885 ‘logarithmic’ equation to the Mack and Seitz data and noticed that it did not fit the Mack and Seitz data well. They therefore proposed a different equation, the sum of two exponentials: $Q(t)=\mu_{1}e^{-a_{1}}+\mu_{2}e^{-a_{2}}$ A similar function has independently been proposed by Rubin, Hinton and Wenzel [39] to successfully fit a forgetting curve with very large numbers of observations per data point, which could not be fitted satisfactorily with any of the more than hundred functions studied in Rubin and Wenzel [2]. None of these authors gave a forgetting mechanism associated with these functions.

Though providing us with a superior fit, a disadvantage of the summed exponential is that there are no memory models that explain why forgetting might have this shape. As remarked by several authors investigating the shape of learning and forgetting [35,38,40], simply fitting sums of exponentials is expected to yield progressively better fits for the simple reasons that any function may be approximated by such a sum, which is related to the Laplace transformation.

A model of forgetting and amnesia developed by our group also yields a summed exponential function, but with a different parameterization [41]. This so called Memory Chain Model assumes that a memory passes through several neural processes or stores, from short-term to very long-term memory. While a memory is (exponentially) declining in intensity in Store 1 (e.g., the hippocampus), its contents is steadily transferred to a Store 2 (e.g., the neocortex) from which it will decline at a lower rate. We still have two exponentially declining stores, as in the summed exponential function above, but they are linked by a memory consolidation process. The decay rates in Store 1 and Store 2 are given by *a*_1_ and *a*_2_, respectively. The initial strength of the memory traces in Store 1 are given by *μ*_1_ and the rate of consolidating the contents of Store 1 to Store 2 is given by *μ*_2_. In experiments with dementia patients and experimental animals, Store 1 may typically be identified with the hippocampus and Store 2 with the neocortex. Lesioning Store 1, will produce a retrograde amnesia gradient that can be modeled by the Memory Chain Model simultaneously with the forgetting gradient of healthy controls [42].

The Memory Chain Model (MCM) equation for type of savings studied here is given by

$$Q(t)=\mu_{1}{\text{e}}^{-a_{1}t}+\frac{\mu_{1}\mu_{2}({\text{e}}^{-a_{2}t}-{\text{e}}^{-a_{1}t})}{a_{1}-a_{2}}$$

The MCM function has the same number of parameters but they are arranged differently. The proof that this equation is a mathematical formalization of the memory consolidation process can be found elsewhere [42]. As can be seen in Table 5, the summed exponential and the MCM function give exactly the same fits, though the parameters differ. The gain of using the MCM function lies primarily in the fact that its parameters can be interpreted more clearly, that it is associated with a type of consolidation mechanism, and that also explains other types of data than the savings function [41–43]. The MCM function assumes a neural system consolidation mechanism [44,45] that has been dubbed the 'Standard Consolidation Theory' [46,47], where the latter authors propose a different theory, the so called Multiple Trace Theory of consolidation. It is here not our goal to evaluate the merits of these theories; we have reviewed these and other theories of consolidation elsewhere [48]. We merely want to apply the MCM equation to these four savings curves and evaluate the goodness-of-fit, viewing it as a conceptual improvement of the summed exponential.

If we compare the MCM equation or summed exponential function to the other functions, this only makes sense if we rely on the *AIC*, which takes into account the varying number of parameters. The MCM function (or double exponential function) fits two of the four curves (Mack and Dros) better than Ebbinghaus’ own equations from 1880 and 1885, it gives about the same fit on the Seitz data, and it does much worse on Ebbinghaus' own data. The average *AIC* is 0.5 less than the average *AIC* for the classic Ebbinghaus functions, which—though it indicates a better fit—may not be considered a meaningful difference; a difference of 2 is considered 'meaningful' [49]. We also fitted a single exponential with only two parameters but this fared far much worse on all data sets, including Ebbinghaus’ data (also see [3]).

Summarizing, Ebbinghaus' data fit his own equations and the power function best. The *AIC* indicates that on average the MCM equation (or summed exponential function) is on average better than all equations considered thus far, where the difference with the power function is 1.5. The difference with Ebbinghaus' own equations is only 0.5 but this is partially because his own data have an exceptionally good fit on his own equations, with a very low *AIC* of about -30.5 (and an extremely high 99.8% variance explained). It is likely, however, that Ebbinghaus actively searched for an equation that achieves such an exceptional fit, which in his eyes was no more than a 'summary' of the forgetting curve (see citation above). This also explains why he has no problems substituting a 'logarithmic' equations for the earlier 'power' equation: it shows a slightly better fit.

Fitting data is always done with a purpose. Ebbinghaus achieved a concise summary of his forgetting data, the power function is a parsimonious description of the forgetting function that shows a good or at least adequate fit in many types of forgetting experiments, and the MCM equation attempts to capture the shape of a hypothetical consolidation process in the brain albeit at the expense of additional parameters. Taking into account these extra parameters, however, does not give a worse fit on the *AIC* and approaches a meaningful improvement over the power function.

### The 24-hour point in Ebbinghaus' forgetting curve

When looking at the shapes of the four curves in Fig 2, savings after 1 day (or 2 days) seems higher than expected. Ebbinghaus [8] notices this as well but merely writes it off as a discrepancy from his fitted curve (see above) that still falls within the error bars ([8], p. 62). He clearly did not trust this data point because in his text from 1885 [9] he reports that he later had replicated this 24 hour data point. The replicated data for this point gave a very similar score, so we must consider it a valid measurement. Jenkins and Dallenbach [50], however, interpreted the discrepancy as an effect of sleep, which motivated them to investigate this closer in an experiment on the effect of sleep on forgetting. They also refer to the forgetting curve by Radossawljewitsch [16], who also found higher savings after both 1 and 2 days (0.689 and 0.609, resp.) compared with after 8 hours (0.474). To them, this is suggestive of a very strong effect of sleep, but Finkenbinder [17] points out that Radossawljewitsch's 8-hour data point may not be reliable, because these lists were all relearned during the afternoon, when there was less rapid learning resulting in fewer savings. He, therefore, suggests using a corrected savings score at 8 hours of 0.66, which is not unreasonable given that Ebbinghaus also corrected his savings scores for time-of-day effects, in some cases up to 13%. Even if savings would be 0.66 at 8 hours, however, the 1 day savings score is still higher than the 8 hour score and the 2 day savings is still higher than what one would expect.

Using free recall and retention up to 8 hours, the seminal study by Jenkins and Dallenbach [50] yielded a positive effect of sleep on recall. This effect has since been replicated many times, for example in recent studies on the effects of different sleep stages on both procedural and declarative memory (e.g., [51,52–56]). Whereas the older studies from the 1970s and before typically confound the sleep manipulation with time-of-day effects or fatigue, this is no longer the case in the recent studies, so that there is now very strong evidence that sleep does indeed have an effect on memory independent of the effects of, say, rest or lack of interference. In some of the sleep-memory experiments cited above, we even see a temporary increase in the forgetting curve, where subjects score *better* than after learning in the days following sleep, but not if they skipped the night of sleep after learning (e.g., [53]). This result—and other studies—suggests that the first night of sleep after learning has a particularly important effect on memory that may continue to evolve for several days afterwards. Such an effect may also be observed in savings curve by Mack and to some extent in the Seitz curve, both show a tendency to increase in savings score for two days following learning.

Given that we can trace the history of research on the effects of sleep on memory to the 24 hour point of Ebbinghaus' forgetting curve, we think it is interesting to evaluate this data point more formally. If we can establish the jump in the curve more formally, it will make a stronger case that the 'true shape' of the long-term forgetting curve has a jump in at 24 hours (or perhaps right after the subject has slept), although we may not conclude from this that the local increase is due to sleep per se, which would require more research is necessary for that.

If we first informally inspect the data points shown in Fig 2 and compare them with the fitted power function, we see relatively less forgetting at either day 1, day 2, or both. In all four panels, at least one of these points is above the fitted power function curve at a distance of at least one standard error. The same is true for the fitted curves of the summed exponential, Ebbinghaus’ 1880 ‘power’ function and his 1885 ‘logarithmic’ function (not shown here). We also see this effect for the Memory Chain Model curve in Fig 3, though somewhat less pronounced (in the Seitz panel, the fitted curve crosses the error bars at 1 and 2 days). The reason for this is that the Memory Chain Model already incorporates the effects of a hypothetical consolidation process. In short, we observe that there is seems to be a memory ‘boost’ in the classic Ebbinghaus’ forgetting curve and its replications. The current body of research on sleep and memory would predict such a boost after one or two nights and attribute it to sleep, though for this particular type of experiments this has to established more firmly in further experiments. Whatever its cause, we can better quantify the visually observed boost by including it in the equations fitted. We, therefore, made a variant of the power function that differs only in the addition of a constant *boost* factor to the savings of the retention intervals of 1 day and higher. This power function with boost is also plotted in Fig 2.

The results are mixed, though on average they suggest a trend towards improvement with a *boost* parameter. The original power function had a average *AIC* of -22, a sum-of-squared-differences (*SSD*) of 0.00932 and explained 98.7% of the variance (see Table 5), whereas adding the boost reduces the *AIC* to -23.6, the *SSD* to 0.00628 and increases the average variance explained to 99.1%, putting its goodness-of-fit on a par with the MCM (or summed exponential) function. A difference in average *AIC* of 1.6 may perhaps be called a trend towards a meaningful difference, though there were large differences between the individual subjects. The *boost* parameter in Table 5 shows the size of the upward jump after 24 hours. We see that for Ebbinghaus, this jump is small (0.030), whereas for Mack it is quite substantial (0.131). The Dros data show no evidence for a boost but these fits are probably influenced strongly by the very low 31 day data point.

## Concluding Remark

In 1880, Ebbinghaus [8] set new standards for psychology experiments, already incorporating such ‘modern’ concepts as controlled stimulus materials, counter-balancing of time-of-day effects, guarding against optional stopping, statistical data analysis, and modeling to find a concise mathematical description and further verify his results. The result was a high-quality forgetting curve that has rightfully remained a classic in the field. Replications, including ours, testify to the soundness of his results.

His method can also be seen as a precursor to implicit memory tests in that certain inaccessible representations, seemingly forgotten, can still be relearned faster compared with others that do not show such an advantage. This is evidence of implicit memory because the subjects may not be consciously aware they still possess traces of the memory representations, which cannot be recalled or recognized but that do show savings. The savings method is still used today as a sensitive method to study the decline of foreign languages in order to assess the true extent of linguistic knowledge retained over a long time [57].

Ebbinghaus [8] also emphasizes the importance of sleep for memory, but these remarks are limited to how low-quality or insufficient sleep may have inflated his own learning times at certain dates ([8], p. 66) and as an explanation for the observed time-of-day effects; he learns faster in the morning than at other times. In other words, he acknowledges the effects of previous sleep on current learning, but he does not admit to the role of sleep in slowing down long-term forgetting. The formal analysis above suggests that the classic forgetting curve is not completely smooth but does show a jump at the 1 day retention interval. Current research on the effects of sleep on memory would predict such a jump, but for this particular type of experiment this remains to be established.
